# Progress towards a methodology for high throughput 3D reconstruction of soot nanoparticles via electron tomography

**DOI:** 10.1111/jmi.12680

**Published:** 2018-01-16

**Authors:** E. HAFFNER‐STATON, A. LA ROCCA, M.W. FAY

**Affiliations:** ^1^ Department of Mechanical Materials and Manufacturing Engineering The University of Nottingham University Park Nottingham NG7 2RD U.K.; ^2^ Nanoscale and Microscale Research Centre The University of Nottingham University Park Nottingham NG7 2RD U.K.

**Keywords:** 2D‐TEM, 3D‐TEM, electron tomography, fractal dimension, soot characterization

## Abstract

The aim of this work is to make progress towards the development of 3D reconstruction as a legitimate alternative to traditional 2D characterization of soot. Time constraints are the greatest opposition to its implementation, as currently reconstruction of a single soot particle takes around 5–6 h to complete. As such, the accuracy and detail gains are currently insufficient to challenge 2D characterization of a representative sample (e.g. 200 particles). This work is a consideration of the optimization of the steps included within the computational reconstruction and manual segmentation of soot particles. Our optimal process reduced the time required by over 70% in comparison to a typical procedure, whilst producing models with no appreciable decrease in quality.

## Lay description

Morphological characterization is an important initial step in developing appropriate strategies to minimize the problems caused by soot emissions. Due to the highly irregular nature of soot particles 3D characterization techniques would assist in better extracting parameters necessary, to potentially understand the role of soot particles on human health, their greenhouse effect via absorption of visible light, degradation of marine habitats and on the tribological behaviour of engine lubricating oils. Due to the lack of depth perception, the deficiency of 3D character of engine soot is not clear to see in 2D‐TEM projections, and only with 3D reconstructions we can truly begin to appreciate, particularly quantitatively, the third dimension of soot structures.

This demonstrates the feasibility of electron tomography for 3‐D characterization of soot nanoparticles; proposing a new optimized methodology to give measurable 3D soot models that are of practical use to industry by reducing time required by over 70% in comparison to a typical procedure.

## Introduction

Three‐dimensional (3D) electron tomography is a well‐established technique for the analysis of complex nanoscale 3D structures. However, much of the optimization to date has concentrated on analysis of materials where one structure is fully characteristic of the whole, and of materials with discrete grey scales in transmission electron microscopy (TEM) images. Neither of these conditions apply to the analysis of soot. The complex and varying 3D nature of soot particles produced in combustion makes 3D analysis both highly desirable, and excessively time consuming with a currently typical tomographic procedure, due to the need to analyse multiple structures to obtain an accurate understanding of the sample.

The production of soot is an undesirable yet unavoidable consequence of burning hydrocarbon fuel in internal combustion (IC) engines. The carbonaceous nanoparticles produced can have a profoundly complex and damaging effect on the environments into which they are released, and as such the amount produced by internal combustion engines is limited by European regulation. The complex fractal‐like morphology of soot nanoparticles plays an important role in determining their exact behaviour, and thus the myriad problems that they cause. This includes their effect on human health when inhaled (Broday & Rosenzweig, 2011), contribution to the greenhouse effect via absorption of visible light (Khalizov *et al*., 2009), degradation of marine habitats (Mari *et al*., 2014) and damage to the lubricating properties of engine oil formulations (George *et al*., 2007). Morphological characterization is an important initial step in developing intelligent strategies to combat the problems caused by soot emissions. The aim of the work reported here has been to make progress towards the development of an optimized methodology for the high through‐put morphology characterization of soot nanoparticles in 3D using electron tomography. It is the belief of the authors that the use of 3D characterization has the potential to offer significant advantages compared to the current state of the art (2D TEM) and if developed appropriately could prove a very beneficial tool for researchers in all areas related to the study of soot and similar nanoparticles.

Soot is produced by internal combustion engines when incomplete combustion takes place in the cylinders. Such conditions arise during routine operation due to the presence of fuel rich zones (La Rocca *et al*., 2015). These areas of nonstoichiometric air‐fuel mixtures form for a variety of reasons, including insufficient fuel vaporization due to low engine temperature, limited fuel‐air mixing prior to injection and the presence of fuel films on the cylinder walls and piston due to the injection strategies (La Rocca *et al*., 2015). Additionally, soot can form from the direct pyrolysis of liquid fuel droplets due to the extreme temperatures (Heywood, 1988). Diesel engines and modern direct injection gasoline (GDI) engines are particularly prone to soot formation due to limited fuel‐air mixing prior to injection, though overall engine efficiency and emissions are improved compared to conventional gasoline engines (Mathis *et al*., 2005; Bonatesta *et al*., 2014). Use of exhaust gas recirculation (EGR) systems has been observed to increase the production of nucleation mode particles in the 10–25 nm range (Zhao *et al*., 2013).

From a chemical mechanism perspective, the most important initial precursor to soot formation is generally considered to be acetylene (Richter & Howard, 2000). This, and other small unsaturated species form early during combustion, and undergo a series of radical and polymerization reactions to form small aromatic species, such as benzene and naphthalene. These molecules continue to grow in size via further similar reactions with smaller molecules and with one another. The larger polycyclic aromatic hydrocarbons (PAHs) of around 1000u (unified atomic mass units) that eventually form are the direct precursors to the first soot nuclei that leave the vapour phase. Physical collision of these 1nm spherical structures, concurrent with continued surface growth reactions with smaller species in the gas phase, produces the characteristic soot primary particles (Richter & Howard, 2000). These larger spherical structures have diameter typically around 10–50 nm (La Rocca *et al*., 2013), and are the structural sub‐unit of the distinctive fractal‐like soot particles. Carbonization occurs due to the high temperature conditions during formation, with the result being that soot particles are almost entirely carbon in their elemental constitution (Richter & Howard, 2000). Oxidation of soot and its precursors occurs throughout all stages of soot formation due to the presence of oxygen in the high temperature environment of the combustion chamber, acting to reduce the concentration and size of any soot structures formed (Richter & Howard, 2000). The balance of these processes and thus the amount of soot formed, depends on the particular operating conditions of the engine.

Soot particles, often referred to as agglomerates or aggregates, have a complex, branched morphology resulting from the random collisions of primary particles. The size of these structures typically ranges from 50–500 nm, though this is strongly dependent on the formation conditions (Clague *et al*., 1999; Virtanen *et al*., 2004; Lapuerta *et al*., 2007; La Rocca *et al*., 2013). The term ‘mass fractal’ is often used to describe soot agglomerates (Rogak & Flagan, 1992; Koylu *et al*., 1995; Neer & Koylu, 2006), due to their resemblance to self‐similar fractal patterns, though self‐similarity in soot is often only true over very limited distances. The internal structures of the primary particles has been investigated and typically consists of an inner core and outer shell arrangement (Ishiguro *et al*., 1997). The inner core will contains several small, spherical or ‘fullerenoid’ carbonaceous structures surrounded by short, distorted layers of graphite‐like carbon. The outer shell consists of an onion‐skin like arrangement of short sections of graphite‐like carbon, typically 2–5 carbon layers thick and ∼3 nm wide (Omidvarborna *et al*., 2015). In a small number of publications, additional amorphous carbon structures have been observed in samples of GDI soot‐in‐oil (Uy *et al*., 2014; La Rocca *et al*., 2015).

The major fraction of the soot produced is emitted from the engine with the exhaust gases, contributing to a number of major problems in the environment. The negative effects of soot inhalation on human health have been widely investigated and it has been observed that the surface area of the nanoparticles is linked to their toxicity (Oberdörster *et al*., 1995). In the stratosphere, soot nanoparticles are involved in complex interactions with radiation (Yon *et al*., 2011), particularly visible wavelengths, resulting in a net positive contribution to the greenhouse effect (Khalizov *et al*., 2009). Again it is seen that the fractal morphology, as measured via the fractal dimension, plays a role in determining the refractive properties of soot in these environments and similarly can be used to understand the Brownian coagulation of soot in atmospheric models (Kajino & Kondo, 2011). A further example of the effects of soot is in its concentration in surface waters (Mari *et al*., 2014), where the surface area of soot particles act as active sites for adsorption of waterborne biological species, altering the microbial balance.

A small percentage of the soot produced in the cylinders becomes entrained within the engine lubricant oil, either via blow‐by gases passing the piston rings (Green & Lewis, 2008) or via a thermophoretic mechanism into the oil film on the cylinder walls (Kittelson *et al*., 1990; Suhre & Foster, 1992). The soot‐oil relationship is extremely complex and as such reports in the literature on the effect of soot on lubricant oil viscosity, friction and on engine wear at times appear contradictory (Heywood, 1988; Chinas‐Castillo & Spikes, 2004; Green *et al*., 2006; Green & Lewis, 2007; Antusch *et al*., 2010; Hu *et al*., 2013; Salehi *et al*., 2016). The particular changes that take place depends not only on the concentration of soot, but also on its morphology (Chinas‐Castillo & Spikes, 2004; Antusch *et al*., 2010). It is known, for example that the surface area of soot nanoparticles correlates directly with increases in oil viscosity (Green & Lewis, 2007). Additionally, the oil formulation, contact type and lubrication regime will all play a role in the changes observed. Depending on the particular combination of contact‐type and the lubricant oil chosen in a study, the presence of soot has been observed to cause both increases and decreases in friction and oil viscosity (Hu *et al*., 2013; Salehi *et al*., 2016) and rates of wear within engines (Salehi *et al*., 2016).

It is clear from these examples that in order to efficiently and competently deal with the numerous problems that automotive soot causes, a thorough understanding of its morphology is needed. This will, in turn, permit a deeper understanding of the behaviour of soot and, through correlating properties to observations with modelling studies, enable effective design of solutions. As such, the characterization of soot is an area of great interest for researchers from various disciplines and many methods have been employed to these ends.

Characterization of soot morphology can be achieved using a number of techniques, of various levels of quality, reliability and experimental difficulty. Techniques such as nanoparticle tracking analysis (La Rocca *et al*., 2014a, b) and dynamic light scattering (Liu *et al*., 2003; Asango *et al*., 2014) (NTA and DLS, respectively) track the Brownian motion of particles in suspension as a means calculate the number concentration and size distribution of particles. Although these techniques can measure many thousands of particles within minutes, they only provide information on the diameter of the hydrodynamic volume a particle occupies. In comparison to the known fractal structures of soot particles this information is clearly very limited and often more detailed methods of characterization are preferred.

Over the last few decades, TEM has been widely used for the detailed characterization of soot nanoparticles (Dobbins & Megaridis, 1987; Megaridis & Dobbins, 1990; Palotas *et al*., 1996; Clague *et al*., 1999; Su *et al*., 2004; Devlin *et al*., 2008; Lapuerta *et al*., 2009; La Rocca *et al*., 2013; La Rocca *et al*., 2015; La Rocca *et al*., 2016). From TEM images a variety of parameters may be measured (volume, surface area, radius of gyration, fractal dimension, skeletal length etc.). However, this currently requires the manual measurement of several hundred particles, significantly increasing the time involved compared with the previous methods. Although the morphological detail is greatly increased compared with simple hydrodynamic diameter size distributions, it is still limited in this regard as they are based upon the measurements of two‐dimensional (2D) images. As such, various assumptions, approximations and empirical correction factors must be applied in order to infer the value of 3D morphological properties. For example, the number of primary particles that constitute an agglomerate is often calculated from the 2D area of a particle in TEM images, first by assuming all primary particles are perfectly spherical and of a constant diameter and then by using semi‐empirical correction factors to account for the overlap of primary particles (Neer & Koylu, 2006). In some cases, agglomerate volume is then inferred simply by multiplying the number of primary particles by the volume of the assumed spherical primary particle, ignoring the overlap of the primary particles (Koylu *et al*., 1997). In particular, empirical correction factors may be problematic when applied to a situation that does not resemble the experimental conditions under which they were deduced. In some instances, properties that can be measured from the 2D projection of the particle, such as radius of gyration and fractal dimension, may simply be assumed to represent the 3D values (Gwaze *et al*., 2006; Lapuerta *et al*., 2006; Sachdeva & Attri, 2008; Schenk *et al*., 2015). In such cases the accuracy of this assumption depends on how well the TEM images portray the 3D nature of the particle, i.e. are more accurate if the particles are not particularly large perpendicular to the TEM grid. The effect of these assumptions and approximations is to add an unknown level of uncertainty to morphological properties measured via 2D means, which may limit our ability to understand how soot morphology impacts its effect on lubricant oil properties.

## Emphasis for 3D characterization

A small number of researchers (Medalia, 1970;, Adachi *et al*., 2007), including the authors (La Rocca *et al*., 2016), have expressed concern over the validity of 3D properties inferred from just a single projection, though in the vast majority of publications this is ignored. The notion is that it can be considered incorrect to only use values measured from a single 2D projection of a particle, given that all possible 2D projections are equally valid representations of the 3D structure and all involve the same lack of depth perception. The apparent morphology of a particle in a projection can change considerably if the angle of projection is altered, and as such the value of any measured morphological properties would also change considerably. Previous work by the authors observed that commonly measured morphological properties could vary by up to 60% for the same particle when projection angles over a ±60° range were considered (La Rocca *et al*., 2016). Work by Adachi also found that 2D‐derived properties are highly sensitive to the angle of projection, with similar levels of variability (Adachi *et al*., 2007).

If every soot particle in a sample showed a similar level of variability with the angle of projection and the contribution of all possible projections is considered (i.e. all projection are equally valid), a large uncertainty becomes associated with traditional single projection characterizations. As such, many more particles than the usual ∼200 must be considered for the calculation of statistically valid means, greatly increasing the time taken for 2D characterization of samples.

Additionally, previous works (including that of the authors) have considered the discrepancy between 2D‐ and 3D‐derived morphological parameters via 3D models of simulated (Rogak & Flagan, 1992; Martos *et al*., 2017) and real soot agglomerates (van Poppel *et al*., 2005; Orhan *et al*., 2016). Van Poppel observed that values of surface area and volume from 2D methods were up to 16 and 125 times larger (respectively) than the real 3D‐derived values (van Poppel *et al*., 2005). Rogak's study of simulated agglomerates observed that fractal dimension was generally underestimated by 10–20% when using 2D methods (Rogak & Flagan, 1992). Our own study found agreement with Rogak's work and additionally that volume could be underestimated by up 40% via 2D methods (Orhan *et al*., 2016). Recently, Martos’ study of a large number of simulated agglomerates found that 2D‐derived radius of gyration underestimated the true values by over 45%, leading to a corresponding overestimation of fractal dimension (Martos *et al*., 2017). Whether 2D methods over‐ or underestimate the real values of morphological parameters depends on the exact methods that were used to derive the values from the 2D‐projections.

It is with this concern over the accuracy of 2D‐based measurements, and with the underlying belief that improved characterization leads to improved knowledge of the soot–oil relationship and in‐turn to improved lubricant design, that the authors have turned to 3D characterization as a potential characterization method. In a situation where it is possible to produce highly accurate 3D volume reconstructions of individual particles, any properties measured from these would be of the greatest detail possible and would greatly reduce or even eliminate the need for unrealistic assumptions and correction factors. Additionally, the issue of multiple valid projections of the same particle is not present, and fewer particles need to be considered for the calculation of accurate and valid mean values.

Whilst a handful of publications have proved that soot characterization through 3D volume reconstruction is possible (van Poppel *et al*., 2005; Adachi *et al*., 2007; La Rocca *et al*., 2016), there remain a major challenge to be overcome before the technique becomes a viable alternative to 2D‐TEM, as the time required to produce the models is currently far too long for a statistically significant number of particles to be characterized.

The individual steps required to produce 3D volume reconstructions via electron tomography are discussed below in the context of soot‐specific issues.

### Acquisition

As for traditional 2D characterization, TEM images of soot agglomerates must be acquired for 3D characterization, so soot samples of interest must be prepared for electron microscopy. Due to the low contrast nature of carbon under the TEM beam soot must be deposited onto support films with as low contrast as possible, such as graphene oxide. Soot‐in‐oil samples must be subjected to a cleaning process the remove the oil residue, as it is a severe contaminant under the electron beam (Shuff & Clarke, 1991). This process includes dilution in heptane, followed by washing with diethyl ether and is described in greater detail in (La Rocca *et al*., 2013). Gold nanoparticle fiducial makers must then be added to the grid to facilitate high quality alignment of images (see next section for further details).

The tomographic reconstruction process is based upon Fourier Slice Theory, which states that the 3D volume of an object can be recreated from the complete set of its 2D projections (Frank, 2008). The use of the term ‘projections’ is an important nuance, and refers to the fact that contrast in TEM images used for tomography should be monotonically varying functions of the mass (i.e. mass‐thickness contrast is dominant in forming the images, as opposed to diffraction contrast etc.; Midgley & Weyland, 2003; Hawkes, 2006). This is known as the ‘projection requirement’, and for soot particles, conventional TEM images adequately fulfil this requirement.

A series of projections is produced by capturing multiple images of a soot particle as the TEM grid is tilted at a series of angles, most commonly in 1–2° increments. In theory the more complete the set of images the more accurate the reconstructed volume, though limitations of the TEM machinery mean that in most cases it is limited to a maximum tilt range of ±60‐70°.

Particles situated close to the edges of the grid can become obscured by the copper mesh at higher angles of tilt, and it may become impossible to collect a tilt series over a sufficient range. So although for 2D TEM characterization particles needed only to be clearly visible, for 3D reconstruction we have additional restrictions on the location of particles, potentially reducing the number of suitable particles significantly. Also, if particles are present in too high concentrations they can obscure one another, and not allow for the reconstruction of individual particles. The transparency of soot nanoparticles under the electron beam means that it often hard to tell whether overlapping agglomerates are indeed individuals or part of a single larger structure.

Once suitable particles have been found, the tilt series of images can be collected. Tilt series can contain as many as 100–200 images, and take up to an hour to complete. The large number of images required exposes the soot samples to high beam dosage, which can be problematic for soot‐in‐oil samples. Residual oil can cause severe contamination of the image, obscuring regions of interest (La Rocca *et al*., 2015), meaning quality tomographic reconstruction is no longer possible.

### Alignment

Prior to the reconstruction process the images in the tilt series must be aligned to a common coordinate system, else the tomogram produced will be of poor quality. Although the software used to capture the tilt series in this work [SerialEM (Kremer *et al*., 1996)] performs an alignment during acquisition, mechanical limitations of the machinery means the tilt is not perfectly smooth. In studies of high contrast species, such as metal nanoparticles, markerless alignments based on simple cross correlation between adjacent images may be sufficient, but the low contrast of soot species means it is unsuitable for this work (Fernandez, 2012). Hence, we have used high contrast gold particles to improve both the automated acquisition alignment, and the alignment by reconstruction software (IMOD eTomo in this work). In the experience of the authors, fiducial marker alignment in eTomo is neither particularly difficult nor time consuming, and primarily automated. The main drawback associated with fiducial marker alignment is additional experimental step that is required in preparing the grids. Not only must an appropriate concentration of soot particles be added, but fiducial markers must also be added in neither too great, nor too small a concentration.

### Reconstruction

Once a suitably aligned tilt series has been created, the reconstruction can then be performed. There are a number of algorithms that can be employed to produce the tomograms, using several software packages.

The algorithms range from computationally cheap and, in theory, less accurate, to more sophisticated and time consuming iterative methods. Weighted back‐projection (WBP) is one of the simplest and most widely used algorithms, and depending on the software, hardware and number of pixels in the tilt series images, can complete in under a minute (Orhan *et al*., 2016). The simultaneous iterative reconstruction technique (SIRT) is another widely used method that involves some degree of back‐projection, and depending on the number of iterations can take several hours to complete (Fernandez, 2013). Newer methods solve the reconstruction iteratively via an algebraic method and can produce high quality tomograms from relatively few images, though require prior knowledge of grey levels in the specimen (Batenburg *et al*., 2009; Batenburg & Sijbers, 2011), and thus are unsuitable for the study of soot. IMOD with eTomo (Kremer *et al*., 1996), TomoJ (Messaoudil *et al*., 2007) and Tomo3D (Agulleiro & Fernandez, 2011) are examples of commonly used and freely available reconstruction software.

An appraisal of the available reconstruction techniques must be performed in order to discover the optimal method. For viability in comparison to 2D‐characterization, the time required for reconstruction is the greatest concern. For example if reconstruction of a single particle took 1 working day, characterization of an entire sample via a statistically relevant number of individuals (100–200 particles) would take around an entire year to complete, which regardless of any improvements to accuracy is simply too long. Additionally, we must ensure that measures taken to improve speed at this stage do not come at the cost of reducing the quality of the reconstructions to the point that they no longer offer greater morphological information than the current 2D methods.

### Segmentation

Due to the low contrast of soot in TEM images, there is subsequently low contrast in the tomogram between soot particle and background support film. Additionally, reconstruction artefacts lead to varying grey levels within areas of particle matter, increasing the difficulty of the process. Since no generally applicable computational method for segmentation has yet come to the fore, manual segmentation is still amongst the most widely used methods (Fernandez, 2012).

Depending on the size of the particle and the resolution in the TEM images, the tomogram can easily be several hundred slices thick. Manual segmentation of this many images can be extremely time consuming, and is probably the most time intensive step in the entire 3D‐characterization process. Segmentation also uniquely defines the particle model that is produced and characterized, and so defines the values of the parameters measured. Accuracy in manual segmentation is clearly of great importance, yet the more accurately the tomogram is segmented, the more time is required.

There are two obvious ways in which the time taken for segmentation could be reduced. The first is to develop a protocol for the postprocessing of tomogram slices so that automated segmentation is possible. Difficulties envisioned with this approach relate to the strong variability in the quality, contrast, degree of artefacts etc. of the tomograms, which depend upon the specimen under study, imaging conditions, reconstruction algorithms used etc. It may not be possible to develop a methodology robust enough to be generally applicable, and may result instead in a method that requires time for the tuning of postprocessing parameters comparable to that needed for manual segmentation. A second method would be to develop the use of interpolation in manual segmentation, i.e. to manually segment every *n*th slice, and interpolate the results in between. This could dramatically reduce the time required for segmentation, whilst retaining the benefits of manual segmentation (applicable to all types of tomogram, low tech etc.). This method is clearly less desirable than a high quality automated process, though represents a more achievable goal in the short term.

### Elongation correction and validation

Although tomographic reconstruction from TEM images can be performed, in practice it is rarely possible to produce the ‘full set’ of projections that is described in Fourier Transform theory. Mechanical limitations mean that a ±70° tilt range is often the most that can be achieved, resulting in a ‘missing wedge’ of information. This causes an elongation and blurring of the reconstructed volume in the direction of the missing information (i.e. the z‐direction, perpendicular to the TEM grid; Fernandez, 2012). Consequently, even if segmentation perfectly isolates all particle matter within the tomogram the resultant particle model will still be erroneous because of the elongation, which will affect measured parameters such as volume and surface area. As the purpose for the development of a 3D‐TEM methodology for soot is to improve the quality of morphology characterization, it is clearly of great importance that the extent of elongation be quantified, and a strategy be developed to ensure 3D‐TEM actually does offer significant improvements compared to the current state of the art.

In 1980, Radermacher proposed a series of equations to evaluate elongation in tomographic reconstruction as a function of the maximum tilt angle, resulting in the following relation (Radermacher & Hoppe, 1980):

exz=α+ sin α cos αα− sin α cos α.

For a maximum tilt range of ±60°, this results in a supposed elongation factor (*e_xz_*) of 1.55 in the z‐direction. Alternatively, the elongation can be determined by the reconstruction of objects of known dimensions. As for real soot agglomerates these are not known exactly, correction must be carried out using alternate structures whose morphology is well defined. The same reconstruction and segmentation procedures must be applied, and the resultant reconstructed models can be compared to the original. On occasion, researchers have attempted this using the gold nanoparticles added as fiducial markers (Mezerji *et al*., 2011). These particles are spherical and can be acquired with well‐defined diameters as small as 5nm. However, they are far removed from resembling real soot agglomerate morphology. In addition they are opaque to the electron beam, meaning that the projection requirement for tomographic reconstruction is not fulfilled in bright field (BF) TEM, meaning HAADF‐STEM must be used (see (Frank, 2008) for further details on the projection requirement). In the BF‐TEM tomography, gold nanoparticles are particularly prone to the effects of fanning artefacts, and at typical resolutions constitute only a small number of pixels (10–20) As such, elongation calculated via these structures are particularly prone to error in their analysis.

Because of such issues with real structures of known dimensions, researchers in the field of electron tomography have analysed reconstruction elongation and artefacts via the use of simulated data sets (Friedrich *et al*., 2011; Ali *et al*., 2012). The example most closely related to our own area of research is Okyay's study of SEM tomography of soot (Okyay *et al*., 2016), which aimed to quantify the effects of missing wedge elongation via reconstruction of a sphere model measuring the equivalent of 100 nm in diameter. Values of volume and surface area from the reconstructed sphere were compared to values from the original model, showing deviations of 0.6% in volume and 7% in surface area. The size of this structure is clearly more representative of real soot agglomerates than the gold nanoparticles, and the computational nature of the model means the appearance of the 2D projections can easily be tuned to match the contrast and noise in real images (Friedrich *et al*., 2011; Ali *et al*., 2012). However, it is known that distortions in tomography are dependent upon the objects shape and size (Frank, 2008), and so for a truly representative and accurate calculation of the elongation, the calibration species should ideally mimic the defining fractal morphology of soot aggregates as well.

Towards these ends, we have devised a method for the creation of simple, soot‐like 3D models and their TEM‐style tilt series in order to assess the extent of elongation to be expected in the tomographic reconstruction of soot nanoparticles. The elongation factor is calculated for multiple 3D models, and a full ‘original versus reconstructed’ morphology assessment is carried out for a select few. This investigation allows us an idea of the scale of the error we can expect during real soot reconstruction, and in future the ability to design a scheme for the correction of the effects of elongation, blurring, fanning and other reconstruction artefacts.

## Experimental setup and sample preparation

Three model systems of soot were used in this work: flame‐generated soot and diesel and gasoline engine soot extracted from oil in the sump. Diesel soot‐in‐oil was drawn from a high‐pressure common rail direct injection diesel engine which was a single cylinder variant of a multicylinder design, see (La Rocca *et al*., 2016) for further details. Gasoline soot‐in‐oil was collected from the oil sump of a modern 1‐L turbocharged gasoline direct injection passenger car driven in city/urban environment after 6000 miles. All soot samples were deposited onto 300 mesh Graphene Oxide TEM support films obtained from EM Resolutions. Flame‐generated (FG) soot was deposited directly onto the TEM grid. As mentioned, soot samples from engine oil need to undergo a cleaning procedure prior to deposition on the grid. Oil samples were diluted in heptane at a ratio of 1:60, then deposited onto the grid before further washing with diethyl ether. Further details of this procedure can be found in previous publications (La Rocca *et al*., 2013; Orhan *et al*., 2016). Colloidal gold nanoparticles in aqueous suspension were deposited onto the grid to aid alignment of images. Gold nanoparticles were obtained from Generon, and are spherical with 10 nm diameter. Soot characterization was carried out at the Nottingham Nanoscale and Microscale Research Centre (nmRC) using a JEOL 2100F TEM equipped with a Gatan Orius CCD camera, operating at 200 kV.

Reconstructions were performed using IMOD with eTomo (Kremer *et al*., 1996), on a Lenovo PC with a 3.50 GHz Intel Xeon E5‐1620v3 processor (8 CPUs) and 32 GB RAM, using a 64‐bit version of Windows 7. The two single‐axis reconstructions were performed on the same tilt series, which contained an image every 1° over a ±60° tilt range (121 images total). The measurement of morphological parameters from the produced 3D reconstructions was carried out using a combination of MATLAB and Fiji (Schindelin *et al*., 2012). Fiji is a distribution of the open‐source image‐processing software ImageJ (Schindelin *et al*., 2015), pre‐installed with a range of plugins and supporting a range of scripting languages to facilitate development of novel image‐processing algorithms. Morphological characterization was carried out as follows: Volume and surface area were determined using the BoneJ plugin for ImageJ (Doube *et al*., 2010), radius of gyration was determined using a self‐produced ImageJ macro, and fractal dimension using the Box‐count algorithm for Matlab (Moisy, 2008).

### Simulated data

Synthetic soot‐like 3D models were created using a self‐produced macro for ImageJ. Variable numbers of primary particle‐style spheres were randomly generated to interconnect and produce structures similar to fractal‐like soot agglomerates. The number of primary particles varied between 10 and 60, as this is most similar to soot‐in‐oil particles that are our primary research interest. ImageJ was also used to create the tilt series of 2D‐projections, and to alter the contrast and add noise such that the projections appear similar to soot in real TEM images.

## Results and discussion

### Reconstruction algorithm

This section of the paper details the investigation into the optimal reconstruction algorithm to use to facilitate high throughput 3D characterization of soot nanoparticles.

**Figure 1:**
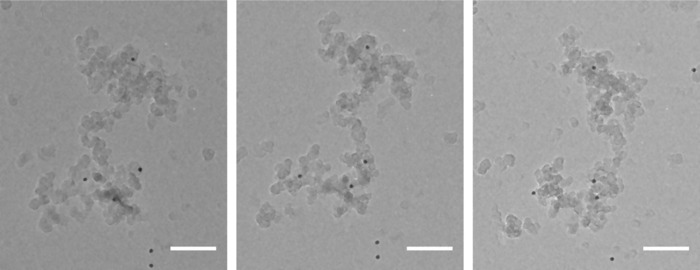
TEM projections of flame‐generated soot particle captured at −30°, 0° and +30° tilt angles. Scale bar equal to 100 nm.

In order to assess the optimal technique, two reconstruction algorithms were used to produce 3D models of the same FG soot particle (Fig. 1). The time required for the computational reconstruction step, and the values measured during the morphological characterization for each method were compared to assess speed and quality. The algorithms employed in this study were single‐axis WBP and single‐axis SIRT (with 50 iterations). WBP is based on a real‐space application of Fourier Transform theory, assuming a monotonic relationship between particle mass and its representation in the projections. SIRT performs an iterative process that compares experimental projections to those computed from an estimated tomogram, and uses back‐projection of the error between them in order to refine the reconstruction (Fernandez, 2012). WBP reconstruction is essentially the lowest quality/computationally cheapest method that may be used, and is expected to be quicker than the iterative SIRT method but produce an inferior tomogram. The tilt series consisted of 121 individual 16‐bit images, 1000 × 668 pixels in size.

The weighted back‐projection algorithm was much quicker than the iterative technique, taking only around 30 s. SIRT reconstruction was performed with 50 iterations, based on the suggestion of Messaoudi *et al*. (Messaoudil *et al*., 2007). After image alignment, the computational step took 135 min to complete. With a view towards high throughput characterization of soot nanoparticles, it appears as though the SIRT method could be too slow regardless of the quality of reconstruction. Our aim is to develop a method to characterize soot samples to a higher level of detail than current methods allow, but within a similar time frame. Characterization of the average morphology of a soot sample requires the consideration of upwards of 100 particles (La Rocca *et al*., 2013), and at this rate would take around 1 month simply for the production of the tomograms, and therefore even longer for the full characterization. Evidently, SIRT must present pronounced improvements in quality in comparison to the WBP method to even be considered.

The quality of reconstruction was assessed by comparing the values of volume, surface area, fractal dimension and radius of gyration measured from each of the 3D particle models. Comparison of absolute accuracy in the reconstruction of the FG particle is not easy because the true values of the morphological parameters are not known. However, assessment of accuracy could be achieved through the reconstruction of a simulated tilt series for a model of known dimensions. Such methods were employed by Okyay *et al*. in their study of SEM tomography of soot and Adachi *et al*. in their electron tomography study of soot via the reconstruction of numerically generated spheres (Adachi *et al*., 2007; Okyay *et al*., 2016). However, in this case absolute accuracy was deemed unnecessary for a comparison of the two methods. Simply put, there was a small enough difference in the values of volume, surface area, etc. that it could be concluded that there was no significant difference in the quality of the reconstructions. The values of the morphological parameters are presented in Table 1.

**Table 1:** Time required for, and morphological parameters measured from two reconstructions of FG soot particle

Algorithm	Reconstruction time (mins)	Z‐depth (1 nm slices)	Volume (nm^3^)	Surface area (nm^2^)	Fractal dimension	Radius of gyration (nm)
WBP	0.5	203	1 819 000	268 900	2.367	148.6
SIRT	135	197	1 920 000	273 800	2.377	148.5

The particle models were produced from the tomogram via manual segmentation. In each slice of the tomogram, the operator differentiated particle matter from background material in the region of interest (ROI) selection using image analysis software ImageJ. Manual segmentation is often preferred in tomogram segmentation as low signal to noise ratio in TEM images renders application of automated/thresholding methods difficult (Fernandez, 2012), particularly due to grey level discrepancy within particle matter. However, automated methods have been employed in tomographic studies of soot (Adachi *et al*., 2007; Okyay *et al*., 2016). Segmentation results in a 3D voxel map of the particle, which was then characterized using a combination of Fiji, the boneJ plugin for ImageJ and self‐produced macros for ImageJ.

**Figure 2:**
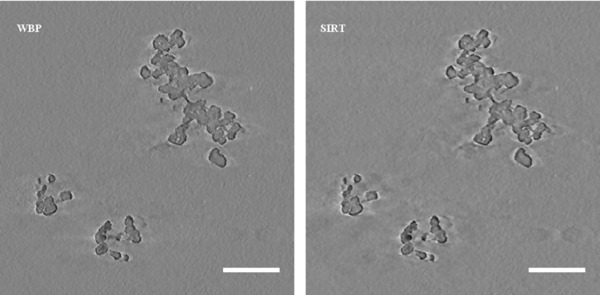
Equivalent slices from within WBP and SIRT tomograms, roughly halfway through the depth of the particle. Note similarity in visual quality and similarity between particle matter in WBP and SIRT tomograms. Scale bars are equal to 100 nm.

In terms of visual quality, there was little difference between the two tomograms. The WBP tomogram was slightly more prone to noise, producing images with a ‘grainier’ quality. Figure 2 shows sections from within similar areas of the tomograms to illustrate this point. As such, the two tomograms were similarly easy to manually segment, i.e. there was a similar degree of subjectivity in distinguishing particle matter from the background.

After segmentation, the content of each of the tomograms were compared by superimposing the ROI sets of one tomogram upon the other tomograms. In doing this, it could easily be seen that the WBP and SIRT tomograms were essentially identical. In every slice the ROI set of one particle appeared to almost perfectly match with the particle matter visible in the other's tomogram. As such, the differences in the measured parameters between these two methods (5.5% in volume, 1.8 % in surface area and <0.5% difference in *D_f_* and *R_g_*) provide us with an example of the amount of error we could consider acceptable as it derives simply from the manual segmentation by the operator, due in part to subjectivity of the particle matter.

**Figure 3:**
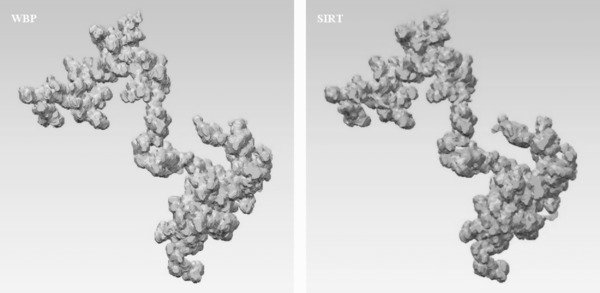
Three‐dimensional models of FG soot particle visualized in UCSF Chimera. Left to right: WBP, SIRT.

It was observed that WBP produces a tomogram not remotely inferior to that produces by SIRT, even with 50 iterations. Given that the former completes so much faster, we can only conclude that WBP is the most appropriate method to use for high throughput reconstruction and characterization. Three‐dimensional renderings of the FG soot particle as produced by WBP and SIRT reconstruction are shown in Figure 3.

### Content of tilt series

Following the findings of the previous section, we continued our investigation into optimization by considering how the number of images contained within a tilt series would affect the speed and quality of 3D reconstruction. Depending on the type of structures being considered and the type of reconstruction algorithm being employed, tomographers use tilt series of varying levels of total tilt ranges and sizes of increments between images (Adachi *et al*., 2007; Okyay *et al*., 2016). Some more advanced algorithms are able to produce high quality reconstructions from as few as 10 images (Okyay *et al*., 2016). A reduction in the number of images can save time during acquisition, but perhaps more importantly can help protect more sensitive specimens from damage caused by excessive beam exposure. With regards to reconstruction of soot‐in‐oil particles, this latter point is quite important, as mineral oil is a severe contaminant under the electron beam (Shuff & Clarke, 1991). In this section, 10 models of the FG soot particle were produced from tilt series that varied in the size of increments between images and the total tilt range (listed in Table 2). Reconstructions were performed using the WBP algorithm in IMOD with eTomo.

**Table 2:** Size of deviations between particles produced from reduced tilt series and FG‐WBP‐1 particle. FG‐WBP‐1 results shown as absolute values, deviations shown as percentage's of FG‐WBP‐1 results. Positive values indicate results larger than those for FG‐WBP‐1 and negative results are those smaller than that for FG‐WBP‐1

Sample	Tilt range	Increment size	Total # images	Volume (nm^3)^	Surface area (nm^2^)	Fractal dimension	Radius of gyration (nm)
FG‐WBP‐1	±60	1°	121	1 819 000	269 000	2.367	148.6
FG‐WBP‐2	±60	2°	61	4.5 %	0.6 %	0.04 %	0.1 %
FG‐WBP‐3	±60	3°	41	1.5 %	−1.3 %	0.45 %	−0.2 %
FG‐WBP‐5	±60	5°	25	−2.6 %	−4.8 %	−0.19 %	0.0 %
FG‐WBP‐50	±50	1°	101	−4.7 %	−4.5 %	0.45 %	−0.9 %
FG‐WBP‐40	±40	1°	81	7.0 %	4.5 %	0.08 %	0.1 %

As in the previous section, the time required for the computational step and the quality of reconstructions via the values of the measured morphological parameters were considered. The results for the WBP algorithm are the focus of this section given the findings of the previous section.

**Figure 4:**
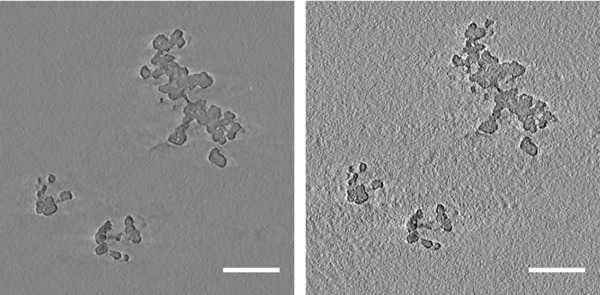
Comparative slices from FG‐WBP‐1 (left) and FG‐WBP‐5 (right) tomograms. Images are not postprocessed.

As WBP reconstruction completed so quickly already (∼30 s for the FG particle), there was no appreciable decrease in time for the ‘reduced’ tilt series. In terms of visual quality, most of the tomograms produced were fairly similar. Only the FG‐WBP‐5 tomogram was of significantly lower quality, with higher levels of noise meaning that some regions of particle matter were less easily discernible from the background in manual segmentation (see Fig. 4). However, as this tomogram was produced from only 25 images in total and by one of the most basic reconstruction algorithms, the quality was still impressive.

As performed in the previous section, the FG‐WBP‐1 ROI set was superimposed on the other tomograms to compare the content/ordering of particle matter. It was observed that the content of the FG‐WBP‐2 tomogram was essentially identical to FG‐WBP‐1 as in every slice the ROI selection was appropriate (as was seen earlier for FG‐SIRT‐1). This meant there were now three particles whose segmentations could be considered as three separate considerations of the same tomogram. The scale of deviations considered acceptable were barely changed by this, as FG‐WBP‐2 possessed an intermediate volume, surface area and fractal dimension. There was a marginal increase in the size of deviations in radius of gyration, though this remained below 0.2%. None of the other tomograms matched the FG‐WBP‐1 ROI set so closely, though differences were fairly minor and overall the particles were very similar.

To assess their quality more quantitatively, particles produced from the reduced tilt series were compared in terms of their morphological parameters to the FG‐WBP‐1 particle (or FG‐SIRT‐1 for the SIRT reconstructions). This was produced from the most complete tilt series and thus the tomogram should be of the highest quality, and the particle should be the most accurate. Large deviations from the values measured for the FG‐WBP‐1 particle would indicate lower quality reconstruction, whilst deviations comparable to those seen between the FG‐WBP‐1 and FG‐SIRT‐1 particles (as discussed in the previous section) indicate a very similar quality.

In terms of this comparison, it was found that almost all of the particles produced from the reduced tilt series showed no deviations from the FG‐WBP‐1 value that were significantly larger than those deemed due only to the manual segmentation process. It was only the tilt series with the smallest tilt range (FG‐WBP‐40) that showed consistently larger deviations, but given that our bounds for acceptable deviations (i.e. those due to subjectivity in manual segmentation) were based on only three sets of results, even these may not be significant. These findings are impressive, and potentially very useful considering that we are able to significantly reduce the number of images, and therefore beam exposure, with no large losses in quality.

There was no clear relationship between the number of images a particle was reconstructed from and how much that particle deviated from FG‐WBP‐1 particle in terms of the morphological parameters, e.g. FG‐WBP‐3 was closest but was produced from second fewest images. Similarly, there was no strong relationship between the sizes of the deviations in the particular parameters. Such behaviour might indicate that when segmenting these tomograms they were similar enough that the limiting factor in quality was the human operator, rather than the appearance of the tomograms.

The results in this section have shown that WBP reconstruction can produce tomograms of good quality from as few as 25 images over a ±60° tilt range. Despite the FG‐WBP‐5 particle showing no significantly larger deviations than any other particle it is the opinion of the authors that the FG‐WBP‐3 protocol, reconstructed from 41 images over a ±60 tilt range, represents the optimal situation as lower image quality for FG‐WBP‐5 hampered segmentation. As the particle with the largest deviations, FG‐WBP‐40, was produced from a relatively large number of images, it appears that reducing the number of images via increasing increment sizes between them is better for particle quality than reducing the overall tilt angle range. The main benefits of these findings is to greatly reduce the beam dosage experienced by the soot particles that are imaged. This will greatly reduce the likelihood of carbon contamination from mineral oil obscuring particles, as was observed in (La Rocca *et al*., 2015), permitting less rigorous sample preparation procedures to be used. Additionally, it will reduce the likelihood of damage or changes to soot structures occurring from beam damage during the acquisition of the tilt series. Despite a reduction in the number of images needed, any improvements to speed of acquisition of a tilt series are expected to be minor as the limiting factor in this case is the tracking of particles between each tilt increment, which takes slightly longer for larger increments sizes.

### Effects of interpolation on segmentation quality

**Figure 5:**
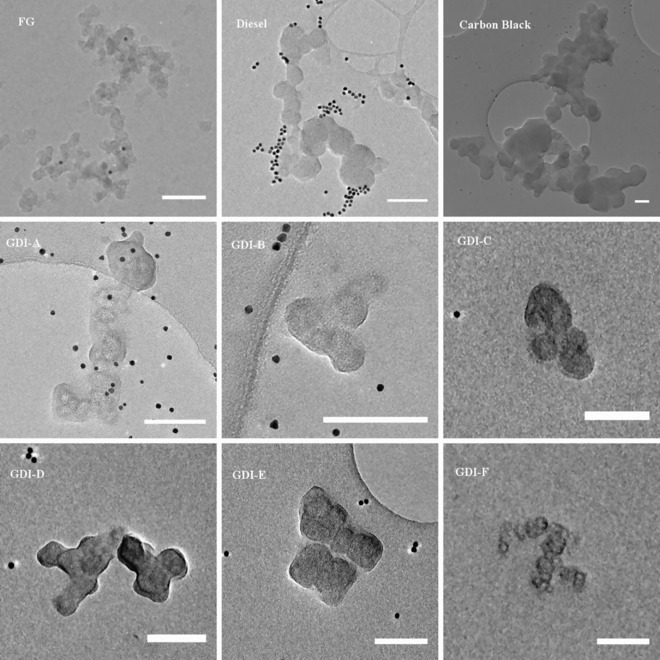
Untilted TEM images of the nine soot particles studied in this section. Scale bar equal to 100 nm.

In an attempt to explore possible improvements to the manual segmentation of soot particles, the effects of differing degrees of interpolated segmentation upon the quality of 3D volume reconstruction were investigated. From the tomograms of nine individual soot particles, multiple versions of each particle were produced by segmentation utilizing five different levels of interpolation. The particles in question come from a range of sources, including the flame‐generated particle considered thus far, carbon black, and soot‐in‐oil from both diesel and GDI engines. Particles are shown in TEM images in Figure 5 below. They represent a diverse range of sizes and shapes, from extensively branched 3D structures over a micrometers in length to highly clustered particles consisting of only a handful of primary particles. Such a set of particles were considered in order to improve the validity of any findings, as to be able to recommend a procedure that may be appropriate for soot particles of any size and morphology. For the FG particle, the FG‐WBP‐1 tomogram was used, whereas all other particles were reconstructed using the WBP‐3 protocol.

For each tomogram, manual selection of the appropriate particle matter was performed on every 20th, 15th, 10th or 5th slice and the intermediate ROI selections were produced via linear interpolation, using ImageJ. Additionally, particles were produced by manually considering the particle matter in every slice, with no interpolated results (termed ‘full’ manual segmentation). Leaving larger increments between manually segmented slices can greatly reduce the time required, but leads to inaccuracies in the size and shape of the produced particle as the interpolation does not make any knowledgeable prediction of the intermediate particle matter. The FG‐WBP‐1 particle already introduced in previous sections represents a full manual segmentation of the tomogram, in which the ROI selection of particle matter is reviewed to be to the operator's satisfaction in every slice.

For each tomogram, the time required for segmentation at each level of detail was recorded, and for the resultant particles the morphological parameters used throughout this work were measured. In this case, the values provided by full segmentation (e.g. the FG‐WBP‐1 and equivalent results for each particle) act as the standard, as they represent the most accurate selection of the particle matter present in the tomogram. Again, deviations from the ‘full’ values were compared to those between the FG‐WBP‐1, ‐WBP‐2 and –SIRT‐1 particles, as this represents the magnitude of deviations that can occur simply due to subjectivity in particle matter and manual segmentation. In an ideal scenario, interpolated segmentation will allow time to be saved during the segmentation whilst still producing a particle that falls within, or close to, the bounds of ‘operator uncertainty’ set by these three particles.

**Figure 6:**
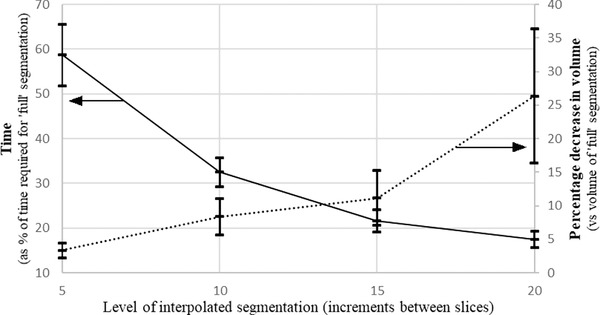
Average decreases in time taken for the various levels of interpolated segmentation, presented as percentage of time required for full manual segmentation (solid line, left y‐axis). Also showing average decrease in particle volume for each level of interpolation, as a percentage of the volume of the fully manually segmented particle (dashed line, right y‐axis). Error bars represent 95% confidence intervals.

The average time required for the different levels of interpolated segmentation is presented in Figure 6, as a percentage of the time that was required for ‘full’ segmentation of each particle. The average deviations in volume at each interpolation level are also presented as percentages of the ‘full’ volume of each particle. Note that all deviations in volume were negative, i.e. all particles produced by interpolated segmentation always had lower volumes in comparison to fully segmented particles.

At each level of segmentation, the amount of time saved was relatively proportional to the number of images that were manually segmented, as would be expected. Of course, improvements to the speed of segmentation are meaningless unless the effects upon the quality of the characterization are also considered. For each of the agglomerates reconstructed, the morphological parameters of the ‘interpolated particles’ were compared to those of the fully segmented particle. The changes in the values of the parameters were quantified as a percentage of the ‘full’ values and the results were averaged to give those shown in Table 3. It should be noted that the values of the various parameters always decreased for interpolated particles compared to the ‘full’ particles, because the particular interpolation method applied with ImageJ had the tendency to underestimate particle matter that was not continuous between adjacent manually segmented slices. The error bars shown are 95% confidence intervals based on the nine different particles considered.

**Table 3:** Decreases in time and morphological parameters at each level of interpolated segmentation as percentage of values for ‘full’ manual segmentation. Values are averages of those for all nine particles

Segmentation Protocol	time required (% of full)	Volume decrease (% of full)	Surface area decrease (% of full)	*D_f_* decrease (% of full)	*R_g_* decrease (% of full)
5^th^	58.7	3.4	3.9	0.08	0.93
10^th^	32.5	8.4	10.7	0.34	2.29
15th	21.7	11.2	15.8	0.42	3.53
20th	17.5	26.4	25.8	0.71	5.21

As mentioned, deviations in the values of the morphological parameters was compared to the size of those observed between the FG‐WBP‐1, ‐WBP‐2 and ‐SIRT‐1 particle. These were: 5.5% in volume, 1.8% in surface area, 0.4% in fractal dimension and 0.2% in radius of gyration. In terms of volume only ‘5th slice’ interpolation produced particles that were within the acceptable limits, with an average decrease of 3.5% compared to fully segmented particles. At an average decrease of 8.4%, 10th slice interpolated presented a more significant reduction in quality. Volume losses up to the ‘15th slice’ level remained fairly linear, but 20th slice segmentation showed a severe drop in quality, with an average decrease of 26% in volume. The data suggested that particles with higher volume/surface area ratios (such as Carbon Black and GDI‐E) lose less volume with interpolation, though ROI selection of the slices took comparatively longer.

The effects of interpolation on the accuracy of the particle surface area were fairly similar. At the 5th slice level, a decrease of 3.9% represented the most acceptable levels of information loss. This was larger than the maximum difference observed between the 3 particles acting as the standard (1.8%), but since there are only 3 results to which we compare they are considered more as guideline than a strict threshold. Losses in surface area at the 10th and 15th slice levels were slightly larger than seen for volume, though at the 20th slice level they were very similar. Again it appeared that particles with higher volume/surface area ratios were less prone to losses in surface area, though as mentioned these particles generally took longer to segment.

Overall, losses in fractal dimension and radius of gyration due to interpolated segmentation were much less pronounced. In the case of the fractal dimension, all levels up to 15th slice segmentation were within the limits suggested by the standards, but even at the 20th slice level the losses are small in absolute terms, at just 0.71% on average (maximum of 1.5% at upper confidence limit). With regards to radius of gyration, interpolated segmentation at any level causes decreases larger than were seen for the three particles acting as our standards. However, in absolute terms the losses are again rather small, limited to 5.2% at the 20th slice level (8.2% at upper confidence interval), and at just 0.9% at the 5th slice level. For these two shape descriptor parameters, it was mostly the particles with a combination of low volume and small z‐depth that had the greatest losses as the level of detail in the interpolated segmentation decreased.

Overall, our data on these nine agglomerates suggested that interpolated segmentation between every 5th slice would provide particles that could be characterized to a level of accuracy similar to that of full manual segmentation. At this level of interpolation, segmentation could be expected to take around 40% less time than a fully manual inspection of the tomogram, concurrent with decreases of around 4.5% in volume, 4.9% in surface area 0.3% in fractal dimension and 1.6% in radius of gyration (all upper limits of the 95% confidence intervals).

The conclusion of our investigations into the optimization of 3D reconstruction is to suggest the following conditions: ±60° tilt series with an image every 3° (41 images total), WBP reconstruction and interpolated segmentation between every 5th slice. By comparison, a typical reconstruction procedure for the study of soot nanoparticles, based on the conditions in the work of van Poppel (van Poppel *et al*., 2005), Adachi (Adachi *et al*., 2007; Adachi & Buseck, 2008), Okyay (Okyay *et al*., 2016) and Cabie (Cabié *et al*., 2016), could be: ±60° tilt series with an image every 1° (121 images total), single‐axis SIRT with 50 iterations and full manual segmentation. Our optimized method was compared to this averaged protocol for reconstruction of the FG soot particle for the time required. In terms of the number of images used, potential beam dosage experienced by the sample is cut by two thirds. This is particularly advantageous for soot‐in‐oil samples, where contamination causing obscuring is a major problem. The time required for SIRT reconstruction (computational step only) of the tilt series was 135 min, compared to <1 min for WBP reconstruction. The time required for alignment etc. prereconstruction was identical. Full manual segmentation of the SIRT tomogram required 144 min, compared to 79 min for segmentation in every 5th slice. Overall, our optimized method reduced the time required by 71.5%.

### Reconstruction of simulated soot structures

**Figure 7:**
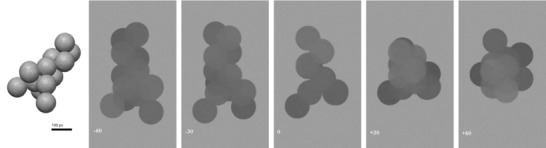
Three‐dimensional rendering of synthetic particle ‘random12’, and TEM‐style 2D projections from the simulated tilt series (tilt axis runs horizontally through the particle in the plane of the page).

For each synthetic structure, three tilt series were generated: ±80° every 1° (161 images), ±60° every 1° (121 images) and ±60° every 3° (41 images) as per the optimal result of the previous section. Each tilt series was reconstructed in order to understand the extent of elongation and quality of reconstruction as a function of tilt range and number of images. Segmentation was carried out manually using ImageJ. Segmented models were characterized using ImageJ and Matlab, as was used for the real‐soot reconstructions. Examples of simulated 3D models and tilt series are shown in Figure 7.

#### Simple sphere reconstruction

To initiate this section of our study, a simple 3D model of a sphere was created in order to be reconstructed, similar to the work by Okyay (Okyay *et al*., 2016). A ±60° tilt series was created for the 350px diameter sphere, and the contrast was altered and artificial noise was added so that it more closely resembled a genuine TEM tilt series. The sphere was subject to WBP reconstruction and full manual segmentation (in contrast to SIRT reconstruction and automated segmentation performed by Okyay). The reconstructed model was 15% longer in the z‐direction with no significant difference in the x‐ and y‐directions, resulting in a lemon‐like shape. The volume and surface area of the reconstructed sphere was 2.3% and 2.4% larger than the original values, respectively. By comparison, Okyay's ±80° (with 10° increments) reconstruction of a 100 px sphere using SIRT with 100 iterations resulted in no elongation, and deviations in volume and surface area of 0.6% and 7%, respectively. The significant deviations between our results is likely due to differences in the sizes of the spheres, reconstruction methods, tilt series content and segmentation method. As already mentioned, it is known that the extent of the deviations due to the missing wedge depends upon the morphology of the object in question, and this sphere reconstruction provides a useful data point to compare the reconstruction of the more complicated models to.

#### Elongation

The ±80° tilt series represents the largest tilt range that can be reconstructed using eTomo. The manual determination of the z‐length of the particles was performed without prior referral to the original z‐length, to limit the effects of bias. The average elongation for the 14 synthetic particles reconstructed from ±80° tilt series was 0.4% ±0.2% (95% CI). In terms of 1‐px thick slices of the tomogram, this is equivalent to at most two slices larger than the original models. At this maximum tilt range, WBP reconstruction apparently results in no significant elongation of the particle matter.

The ±60° tilt series represents the tilt range that can most typically be achieved in routine soot sample analysis. At this tilt range, the average elongation was 4.0% ± 1.0% (95% CI). This is clearly much smaller than seen for the simple sphere reconstruction and the prediction from Radermacher's equation. Elongation has dependency upon the shape of the specimen, as soot‐style synthetic particles were much less affected than the simple sphere (15% vs. average 4%). Additionally, variability in the shapes of the 14 soot‐style particles is reflected in the relatively large confidence interval (±1.0%).

The ±60° with 3° increments tilt‐series represents the fewest images from which good quality tomograms can be produced, as per the results of the previous sections. Unsurprisingly, the measured elongations are very similar to those of the 121 image ±60° tilt series, being on average +3.8% ± 0.8% (95% CI). As seen previously, the degree of noise in these tomograms did increase, though was not severe enough to significantly hamper the segmentation process. Based on the similarity of the synthetic particles to real soot agglomerates, we can be reasonably confident that the extent of elongation in real situations is similar to that seen in this work.

#### Full morphological analysis

**Figure 8:**
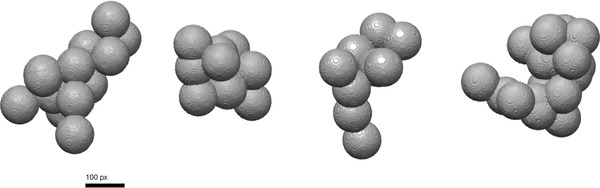
Three‐dimensional renderings of particles (left to right): random12, random2, random5, random7. Generated using UCSF Chimera (Yang *et al*., 2012).

Full morphological analysis was carried out on simulated particles named: random2, random7, random9 and random12. Models are shown in Figure 8 and results detailed in Table 4.

**Table 4:** Size of the deviations in morphological parameters of reconstructed structures in comparison to the original models. X, Y, Z_max_ refer to the maximum distance in those respective direction (Z perpendicular to and X and Y parallel to the tilt axis)

	Percentage deviations versus original values						
Particle	X_max_	Y_max_	Z_max_	Volume	Surface Area	*R_g_*	*D_f_*
Random2	−0.40	0.95	2.92	25.69	−2.29	0.76	2.655
Random7	0.37	0.30	4.43	26.96	0.61	−1.88	2.651
Random9	0.31	0.54	4.23	24.42	1.64	0.98	2.670
Random12	1.34	0.78	2.72	36.17	2.76	−1.18	2.668

**Figure 9:**
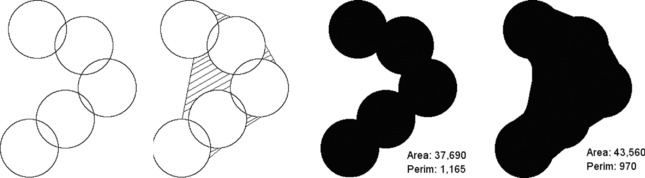
Side‐on representative of structure formed from primary particle‐style spheres, with areas prone to blurring highlighted. Area and perimeter values (in pixels) shown for perfect segmentation and blur‐affected segmentation, showing increase in area (*cf*. Volume in 3D) and decrease in perimeter (*cf*. surface area in 3D).

Generally, deviations in the maximum x and y dimensions of the reconstructed particles were insignificant, with only a single result >1%. The size of the elongation for each particle is shown in the Z_max_ column, and again shows the variation of elongation with particle morphology. Despite only minor increases in the maximum dimensions of the particles, there is clearly a significant overestimation of particle volume resulting from reconstruction and segmentation. The significant thickening of the particle is likely due to reconstruction artefacts resulting from the lack of ‘information’ in the missing‐wedge and between the projections. Elongation, noise and fanning artefacts reduce the definition of particle edges which can cause them to blend together in appearance, particularly in the direction of the missing wedge (Fernandez, 2012). Consequently, some areas of the tomogram falsely appear to contain particle matter and there is an overselection in the segmentation. These effects are particularly prominent on shielded/internal surfaces, especially those towards the middle of the structures and act to increase the particle volume and reduce the surface area, as demonstrated from a 2D viewpoint in Figure 9.

In practice, agglomerate volume is not of much use individually and parameters like surface area and fractal dimension offer a more interesting account of the fractal morphology and are more relevant to the behaviour and effects of the particles. For those such parameters, reconstructed volumes fare much better at in comparison to the true values. Variation in surface area, an important parameter known to relate directly to cytotoxicity (Oberdörster *et al.*, 1995), greenhouse effects (Khalizov *et al*., 2009), and engine wear and friction (Green & Lewis, 2008), is rather small (<3%). Similarly, radius of gyration and fractal dimension measured from reconstructions are within 2% of the original values, implying that the particles retain their particular fractal identity after reconstruction. If these results are indeed representative of those for real soot agglomerates (as we have tried to the best of our abilities to make so, with mimic of fractal geometry etc.) it represents an extraordinary level of accuracy, especially in comparison to the compounding of known and unknown errors in 2D‐TEM characterization that was described in earlier sections.

### Three‐dimensional soot‐in‐oil structures

Although soot characteristics are impacted by fuel‐type, injection strategy and operating conditions, some key similarities and differences between flame generated, GDI and diesel soot have emerged from this study. The improvements to the efficiency of the 3D morphology characterization procedure detailed in this paper mean that it has been possible for the authors to begin to characterize multiple soot structures from particular soot samples in a timely manner. Whilst a sample large enough for statistically relevant characterizations (e.g. >200 individual agglomerates typically) has not yet been reconstructed, a sufficient number have been to allow a qualitative understanding of the typical 3D characteristics of agglomerates from different samples. So far, carbon black, flame‐generated soot, diesel soot‐in‐oil and gasoline soot‐in‐oil samples have been considered.

Typically, flame‐generated soot and carbon black nanoparticles showed an extensive, fractal, 3D structure, i.e. particle matter extended in all three dimensions from the centre, in the standard structure of agglomerated spherical primary‐particles.

Diesel soot‐in‐oil particles were also fractal aggregates of primary particles. However, in contrast to the flame‐generated soot they were notably 2D, in general being just a single primary particle thick in the z‐direction (perpendicular to TEM grid).

**Figure 10:**
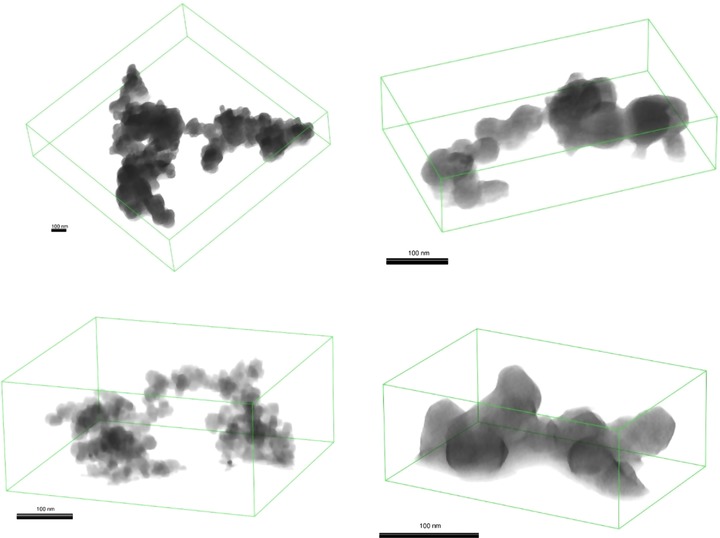
Examples of typical structures of flame‐generated, carbon black, diesel soot‐in‐oil and GDI soot‐in‐oil. In each image, the z‐direction is perpendicular to the scale bar (vertical), as the particles are being viewed from the side on. GDI‐D and diesel soot structures are primarily just 1 primary particle thick in z‐direction. Scale bar equal to 100 nm in each image.

The structure of soot agglomerates in the GDI drain oil was also fairly typical in terms of a fractal arrangement of primary particles. Similarly to diesel soot‐in‐oil, the structures were fairly small and their extension in space primarily limited to just two dimensions. However, GDI soot‐in‐oil structures weren't quite as 2D, as structures were generally at least two primary particles thick in the z‐direction in some sections of the particle. Examples of 3D models of soot structures from these different sources are shown in Figure 10.

Certainly such observations are qualitative, but are a promising indication of the type of extra information 3D morphology characterization can offer. Due to the lack of depth perception, the deficiency of 3D character in diesel and GDI soot‐in‐oil is not clear to see in 2D‐TEM projections and only with 3D reconstructions we can truly begin to appreciate, particularly quantitatively, the third dimension of soot structures. The gasoline soot has smaller primary particles, typically 30 nm, than carbon black (mean 48 nm) but larger than diesel soot (mean 20 nm; La Rocca *et al*., 2013).

Flame generated soot and carbon black are often used to synthetically contaminate lubricants. It is worth mentioning that the FG particle considered in this study showed a high morphological complexity; we estimate that *n_po_* = 222, *via* 3D volume divided by the assumed spherical, 25 nm diameter primary particle volume. Typically only 4.5% of the agglomerates sampled from diesel exhaust are composed of more than 200 primary particles (Martos *et al*., 2017). This raises a question over the use of CB and FG soot as a surrogate for engine soot.

## Conclusions

Highly detailed characterization of soot nanoparticles can help to more fully understand soot formation and its health and environmental implications. Due to their size, TEM has been the most widely used method to achieve this. TEM produces 2D projections of soot agglomerates and the lack of depth perception leads to underestimation of commonly measured morphological parameters. High quality 3D reconstruction of soot agglomerates would eliminate this error. However, 3D study of soot aggregates is in its infancy and a major barrier to the success of the technique is its speed.

Three‐dimensional volume reconstruction of flame‐generated and soot‐in‐oil nanoparticles was successfully achieved through the use of bright‐field TEM. Optimization of 3D‐TEM methods allows for high‐throughput soot nanoparticles analysis. In comparison to a typical procedure, our optimized methodology reduced the time required by over 70% and cut beam exposure by two thirds with no appreciable decrease in the quality of the produced 3D model.

WBP and SIRT reconstruction algorithms generated tomograms of good quality with no significant difference in morphological parameters; the WBP is considerably quicker than the iterative technique. Interpolated segmentation between every 5th slice allowed for a level of accuracy similar to that of full manual segmentation; with a 40% reduction in time.

Flame‐generated particle and carbon black under study showed an extensive 3D structure; on the contrary, the diesel soot‐in‐oil particle was notably 2D, being just a single primary particle thick in the z‐direction. Gasoline soot‐in‐oil structures were generally at least two primary particles thick in the z‐direction in some sections of the particle.

Carbon black showed a mean primary particle diameter of 48 nm, whereas gasoline soot was on average typically around 30nm and larger than diesel soot.

Synthetic soot‐like agglomerates and their TEM‐style tilt series were generated using image processing software ImageJ. These structures were subjected to WBP reconstruction on tilt‐series of various compositions. The average elongation factor for 14 synthetic structures was calculated as 0.2% for ±80° tilt series and 4% for ±60° tilt series. In terms of morphological properties, volume was significantly overestimated due to the effects of reconstruction artefacts causing over‐selection in segmentation. However, values of surface area, radius of gyration and fractal dimension were close to the original values (<3% deviation).
